# Static and Dynamic Loading Behavior of Ti6Al4V Honeycomb Structures Manufactured by Laser Engineered Net Shaping (LENS^TM^) Technology

**DOI:** 10.3390/ma12081225

**Published:** 2019-04-15

**Authors:** Anna Antolak-Dudka, Paweł Płatek, Tomasz Durejko, Paweł Baranowski, Jerzy Małachowski, Marcin Sarzyński, Tomasz Czujko

**Affiliations:** 1 Department of Advanced Materials and Technologies, Military University of Technology, Urbanowicza 2, Warsaw 00-908, Poland; tomasz.czujko@wat.edu.pl; 2 Institute of Armament Technology, Military University of Technology, Urbanowicza 2, Warsaw 00-908, Poland; pawel.platek@wat.edu.pl (P.P.); marcin.sarzynski@wat.edu.pl (M.S.); 3 Department of Mechanics and Applied Computer Science, Military University of Technology, Urbanowicza 2, Warsaw 00-908, Poland; pawel.baranowski@wat.edu.pl (P.B.); jerzy.malachowski@wat.edu.pl (J.M.)

**Keywords:** honeycomb structure, additive manufacturing, laser engineered net shaping, LENS, Ti6Al4V alloy, energy absorption, dynamic tests

## Abstract

Laser Engineered Net Shaping (LENS^TM^) is currently a promising and developing technique. It allows for shortening the time between the design stage and the manufacturing process. LENS is an alternative to classic metal manufacturing methods, such as casting and plastic working. Moreover, it enables the production of finished spatial structures using different types of metallic powders as starting materials. Using this technology, thin-walled honeycomb structures with four different cell sizes were obtained. The technological parameters of the manufacturing process were selected experimentally, and the initial powder was a spherical Ti6Al4V powder with a particle size of 45–105 µm. The dimensions of the specimens were approximately 40 × 40 × 10 mm, and the wall thickness was approximately 0.7 mm. The geometrical quality and the surface roughness of the manufactured structures were investigated. Due to the high cooling rates occurring during the LENS process, the microstructure for this alloy consists only of the martensitic α’ phase. In order to increase the mechanical parameters, it was necessary to apply post processing heat treatment leading to the creation of a two-phase α + β structure. The main aim of this investigation was to study the energy absorption of additively manufactured regular cellular structures with a honeycomb topology under static and dynamic loading conditions.

## 1. Introduction

Currently, the progress of civilization forces scientists and engineers to discover new technologies and materials necessary to optimize the products used in all areas of life. The automotive and aviation industries still require new solutions in terms of safety, where a strong impact is placed on the elements used so that they possess high energy absorption capacity during crash tests [1,2,3,4]. Moreover, these requirements are also essential in relation to military applications, such as passive protective systems [5,6] and various kinds of critical infrastructure elements [7]. There are many solutions based on lightweight cellular structures made from different materials, i.e., tubes, sandwiches, or honeycombs, which have been studied under static as well as dynamic loading conditions [8,9,10,11,12,13]. Particularly noteworthy is honeycomb structure topology, which is popular as an energy absorber; it also has received great attention in the biomedical field for applications such as 3D porous scaffolds for tissue engineering and its regeneration [14,15,16,17]. The phenomena of this structure result from its original properties. It is a combination of a low value of relative density and a high stiffness that derives from geometry and allows for the minimization of the amount of material used. Additionally, the periodic arrangement of cells plays a significant role. It has been observed that the crush strength depends on cell shape (hexagonal cells with different branch angles were studied) [18,19,20,21,22] and wall thickness [23,24,25,26]. The honeycomb structures were investigated with numerical analysis or experimentally in both in-plane [27,28,29,30,31] and out-of-plane directions [20,32,33,34,35,36] to obtain various parameters to provide answers about the mechanisms of failure. 

Cellular structures exist as regular and stochastic objects. Stochastic objects are mostly manufactured by conventional methods such as vapor deposition, casting, sintering, and foaming polymer or metallic materials [37,38]. Unfortunately, there are some difficulties associated with these techniques, such as insufficient precision of cell projections, porosity of the produced structures and anisotropy of mechanical properties that derive from the stochastic arrangement of cells and differing unit cell sizes. Researchers have been looking for new technological solutions to overcome these limitations. One of the rapidly developing routes in manufacturing technologies is additive manufacturing (AM), which enables the production of regular cellular structures [39,40]. It is also called rapid prototyping (RP), which can be used as an original method for the fabrication of elements with periodically spaced cells. There are several different techniques that direct the fabrication of component parts by building them layer by layer with the use of various types of metal alloys. Generally, these methods are divided in two categories: Powder Bed Fusion (PBF) and DED (Direct Energy Deposition) [41]. The first one is represented by SLM (Selective Laser Melting), (DMLS) Direct Metal Laser Sintering, and EBM (Electron Beam Melting). Mentioned systems are very popular and allow for manufacturing regular structures with a complex geometry and a very low mass. The main idea of working this type of system is described in detail in following papers [40,42,43]. The other DED group of Metal Additive Manufacturing system is represented by the Laser Engineering Net Shaping system. It was discovered at Sandia National Laboratories and commercialized by Optomec, Inc. in 1997 [44]. It is a technique that allows for saving processing time by shortening the period between the design stage and the manufacturing process. The building of near-net shaped components can be fully controlled, and this is one of the most important benefits. The amount of given powder, the feed of the work table, the laser power and the focus of the laser beam can be precisely selected depending on the needs. The completed 3D parts are made by building them layer-by-layer from powder applied directly to the place the laser beam affects. Components manufactured by the LENS technique can be made from easily accessible engineering materials such as stainless steel, Ni-based super alloys or titanium alloys. The mechanical properties of parts made by the LENS technique have been presented in following scientific papers [37,45,46,47]. The microstructure, mechanical properties and corrosion resistance of 316L stainless steel samples manufactured by the LENS technique were investigated by Ziętala et al. [48]. These samples were characterized by unique dual-phase microstructures unprecedented in stainless steel fabricated by conventional methods, which is the reason for the improvement of the mechanical and corrosion properties. There are also a few works concerning the components made from titanium alloys using the LENS technique. The relationship between the influence of building parameters and deposition of Ti6Al4V samples was investigated by Kummailil [49]. Furthermore, Zhai et al. conducted small and long fatigue crack growth tests [50]. In the results, it was shown that the lamellar structure that is created during post-LENS heat treatment offers higher fracture toughness and ductility. Blackwell et al. examined the possibility of the production of alpha-beta Ti6Al2Sn4Zr6Mo (Ti-6246) titanium alloy samples [46]. Additionally, materials with a chemical gradient obtained by the LENS technique are becoming more focused. There are many different combinations of materials used, i.e., TiC/Ti composite with compositions changing from pure Ti to 95 vol.% TiC [45] or thin wall tubes with a Fe_3_Al/SS316L graded structure [51]. 

On the basis of a conducted literature review, the authors have spotted that there is a limited number of papers considering the possibility of using the DED LENS system in the manufacturing process of regular cellular structures with high energy absorption capacity. Results of investigations presented in many papers are limited to simple geometrical shapes of absorbers like cylindrical and rectangular tubes. Moreover, many of them were realized with the use of stainless-steel powder alloys which give good-quality manufactured models. Titanium alloy seems to be the perfect candidate because of its significant properties of high strength-to-weight ratio, excellent corrosion resistance and high melting point. All these advantages make this alloy very attractive for applications in the medical, aviation, and aerospace industries. The Ti6Al4V chemical composition is the most popular among titanium-based alloys, and it is very popular as a material used in additive manufacturing techniques. The mechanical properties of this alloy closely depend on the structure and size as well as the morphology of the grains. At equilibrium, Ti6Al4V is an alloy consisting of two phases: α and β phase. However, additive manufacturing techniques are characterized by the fact that during production of the details, the degree of cooling rates is so high whereby a martensitic α’ phase creates. The presence of this phase increases the yield strength and tensile strength with a simultaneous decrease in the plasticity of the built samples. In order to improve the ductility or the fracture toughness a post-processing heat treatment is required and provides a two-phases structure [43,52,53]. The heat treatment applied for the components manufactured using additive techniques from various materials can also improve the microstructure homogeneity or removes stresses created during the building process [54,55]. 

Taking into account the mechanical properties of Ti6Al4V titanium alloy and the technological possibilities of the LENS system, the authors proposed investigations to examine the possibility of obtaining Ti6Al4V regular structures with hexagonal cells by the LENS technique. Mapping quality, metallurgical quality and microstructure were tested for the structures before and after applying the appropriate heat treatment. This paper is a continuation of the authors’ previous paper [56] in which a methodology investigation of the deformation process of honeycomb cellular structures manufactured using LENS was discussed. Additionally, the mechanical response of manufactured specimens of structures were evaluated not only in static, but also under dynamic loading conditions.

## 2. Materials and Methods

### 2.1. LENS^TM^ Technique

**Figure 1:**
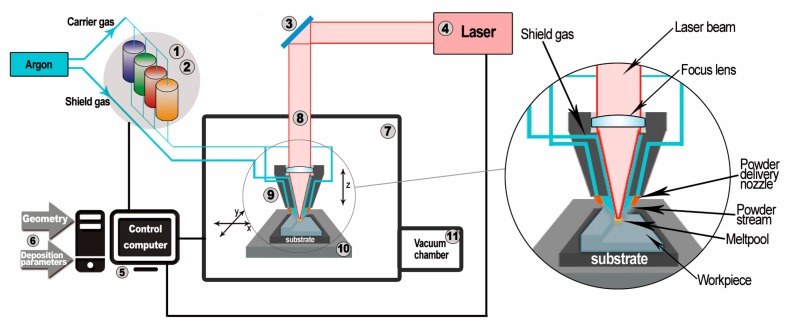
Scheme of the laser engineered net shaping (LENS) system [56]: **1**. Powder supply; **2**. pneumatic vibrating system; **3**. optical system; **4**. IPG fiber laser; **5**. controlling computers; **6**. input data; **7**. working chamber; **8**. optical path of the laser; **9**. working head with four nozzles; **10**. numerically controlled working table (movement in the *X*-*Y* plane); **11**. antechamber.

The components presented in this work were manufactured by the Optomec LENS MR-7 (Albuquerque, USA). The scheme of the LENS system operation is shown in Figure 1. In general, the principle of the device’s operation is based on the selective deposition of metallic or ceramic powders on a prepared substrate or on the previously built layer and melting of them with 500 W of high-power fiber laser at the same time.

The LENS machine has an operating system that allows the operator to design and prepare the manufacturing process. The building of components starts with a 3D CAD solid model, which is sliced by the PartPrep program into layers of an assumed thickness. In the next step the LENS control software selects and determines production parameters, such as the powder flow rate, acceleration, and deceleration of the working table or laser power. 

Powders used in this technique should be of high purity and chemically homogenous with a spherical shape. The recommended particle size distribution ranges from 45–150 µm [57]. Meeting these requirements will ensure good metallurgical quality of the produced components. Four chemically different powders can be used simultaneously during one technological process since the device is equipped with four powder feeders. In addition, the feeding of powders from nozzles placed in the laser head is very precise, even if the quantity of the powder’s flow is small. This allows for creating gradient structures or structures reinforced with the strengthening phase. The substrate on which the previously designed element will be built should have the same chemical composition as the batch powder. This will avoid the occurrence of very high stresses that can be generated during the process.

The LENS process is carried out under an argon gas atmosphere; therefore, the amount of oxygen molecules in the chamber is below 10 parts per million. For this reason, powders with high reactivity can be used in the production process. 

### 2.2. Modelling of Thin-Walled Honeycomb Structure

**Figure 2:**
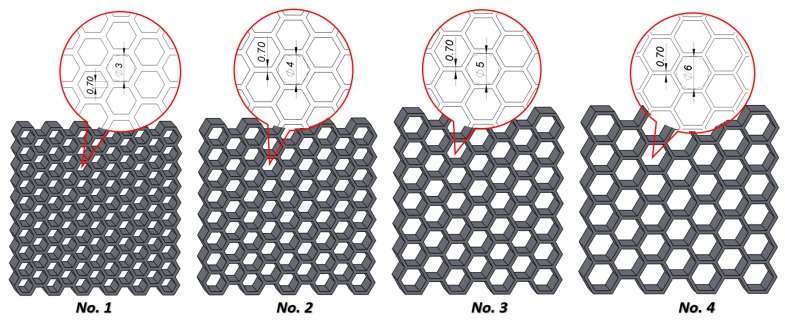
Four variants with of thin-walled honeycomb structures differing in the size of the cells (No. 1–No. 4, dimensions in mm).

The aim of this work was to produce four variants of thin-walled honeycomb structures differing in the size of the unit cells. The selection of the cell sizes was a result of the structure’s geometrical optimization and the technological capabilities of the device. It was determined that the smallest diameter of a circle described on a hexagonal cell possibly obtained by the LENS technique is 3 mm (Figure 2). The other three cells are 4, 5, and 6 mm in diameter. The assumed dimensions of the obtained structures were approximately 40 × 40 × 10 mm, and the wall thickness should be approximately 0.7 ± 0.1 mm. The value of the relative density obtained for the specimens is presented in Table 1. 

**Figure 3:**
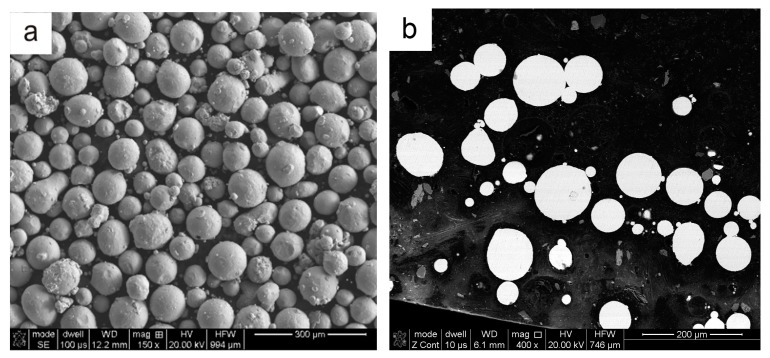
The morphology (**a**) and microstructure (**b**) of the Ti6Al4V powder used for manufacturing of honeycomb structures by the laser engineered net shaping (LENS^TM^) technique.

For manufacturing the above-presented structures, a commercial Ti6Al4V powder was used. The powder was produced by an argon atomization method and was delivered by TLS Technik GmbH & Co. The size of the powder particles ranges from 45–105 µm with a spherical shape. The morphology and microstructure of the powders are given in Figure 3. It has been confirmed that the particles have a spherical shape and mostly smooth surface with microsatellites. In the cross-section, some pores with micrometer size were observed inside the particles.

The honeycomb structures were built using the laser engineered net shaping (LENS^TM^) technique on a Ti6Al4V substrate that was previously skimmed with acetone and sandblasted. The process was conducted in an argon atmosphere, and the oxygen content in the working chamber was approximately 2 ppm. The parameters of the manufacturing process were selected experimentally, and they are presented in Table 2.

**Figure 4:**
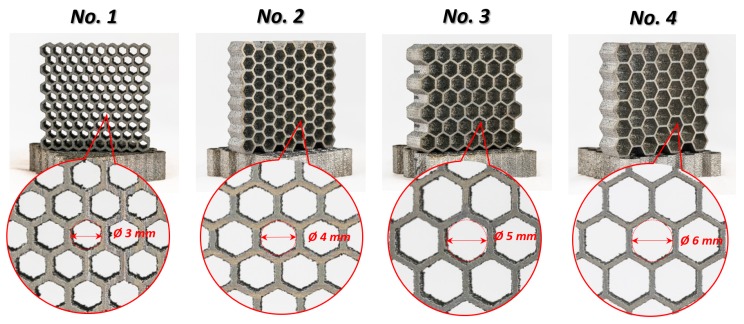
Four variants of Ti6Al4V thin-walled honeycomb structures with different the cell size (No. 1–No. 4) manufactured by the laser engineered net shaping (LENS^TM^) technique.

The obtained thin-walled structures presented in Figure 4 were examined in terms of their geometrical and microstructural properties. 

## 3. Results and Discussion

### 3.1. Geometrical Assessment

**Figure 5:**
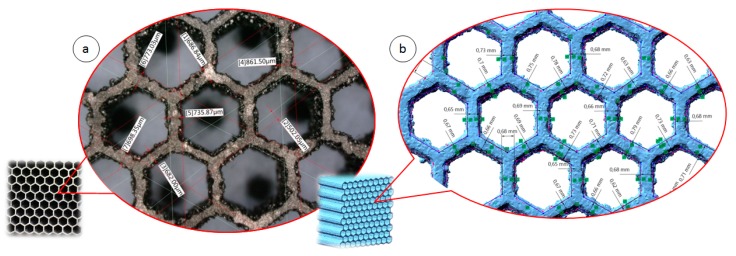
Geometrical evaluation of structure specimens: (**a**) with the application of optical microscopy, (**b**) based on a 3D model reconstructed from Computed Tomography (CT) data [56].

The geometrical quality of the manufactured structure specimens was evaluated based on the data gathered with the application of computer tomography Metrology XTH 225 (Nikon, Leuven, Belgium) and photographs made with the use of optical microscopy (VHX-6000, Keyence International NV/SA, Mechelen, Belgium). The results of the measurements are presented in Figure 5, and they were partially presented in [56]. The obtained wall thickness of specimens differ in comparison to the values assumed during the preparation of the 3D CAD model. It is caused mainly due to the adopted regime of the structure manufacturing process. The wall thickness was defined as a single route of the working head.

The honeycomb structures were subjected to heat treatment just after manufacturing, which was performed at 1050 °C for 2 h. The process was undertaken in a Nabertherm R80/b750/12-B170 tubular furnace (Nabertherm GmbH, Lilienthal, Germany) with a low vacuum. The furnace chamber was purged with argon before the heating processes started. The specimens were heated and cooled down with the furnace. The choice of heat treatment parameters was determined by the analysis of literature [58,59].

**Figure 6:**
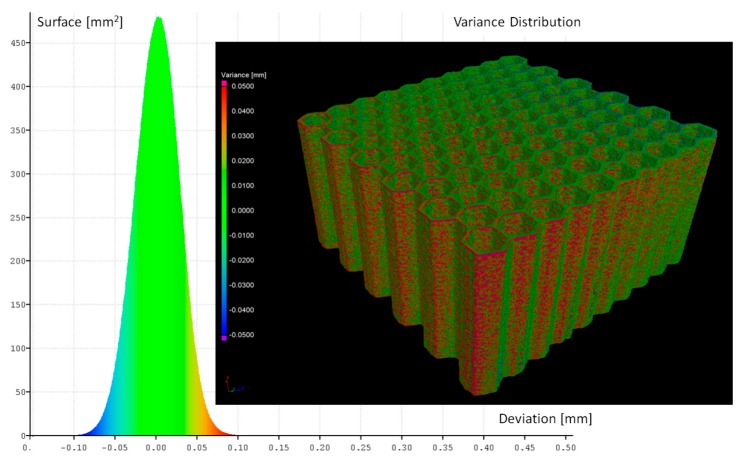
Geometrical quality control of specimen No. 1 before versus after heat treatment.

**Figure 7:**
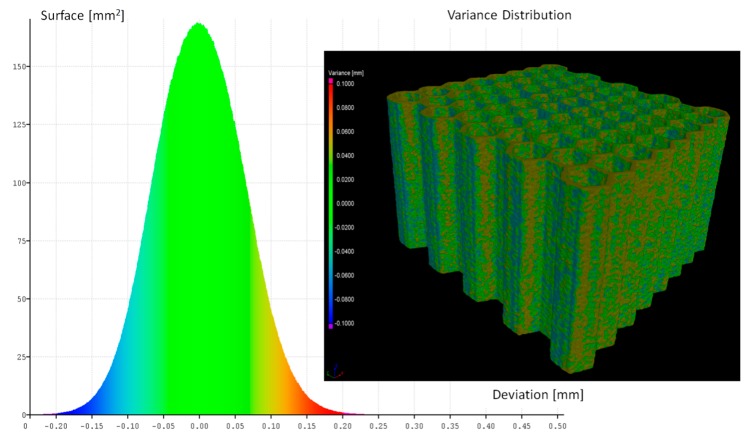
Geometrical quality control of specimen No. 2 before versus after heat treatment.

**Figure 8:**
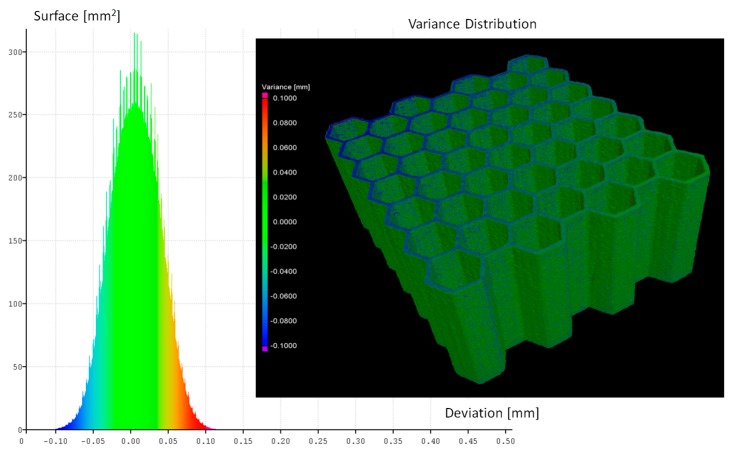
Geometrical quality control of specimen No. 3 before verses after heat treatment.

**Figure 9:**
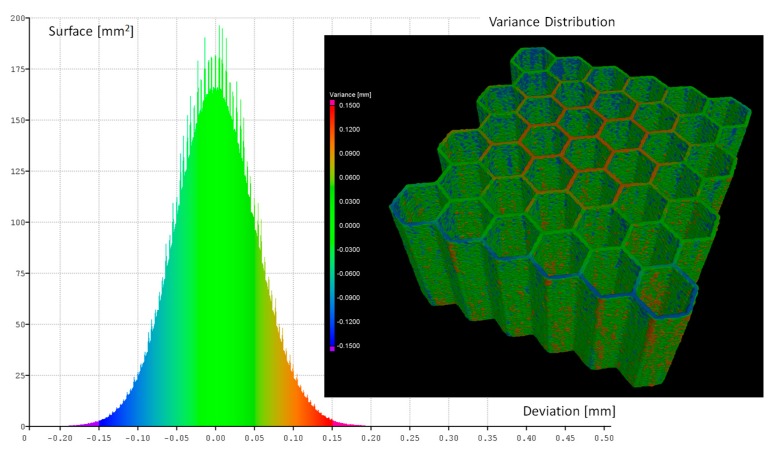
Geometrical quality control of specimen No. 4 before versus after heat treatment.

The influence of the adopted heat treatment process on structure geometrical quality was evaluated based on the analysis of CT data collected before and after the process. Based on the comparison of the obtained results, spatial maps of the geometrical deviation for all specimens were defined (Figure 6, Figure 7, Figure 8 and Figure 9). 

Based on the presented figures, it could be stated that the additional applied heat treatment process strongly influenced the stress relief process. The lowest value of the elementary unit cell size and the highest value of the relative density of specimen No. 1 indicated the highest surface dimensional deviation (Figure 6). It was mainly caused by the poor heat dissipation process, which also affected the structure of the material. Implementation of a larger value of unit cell size enabled better geometrical quality. Additionally, due to the lower value of relative density, the thermal conditions of heat dissipation were better and caused the lowest deviation of structure dimensions (Figure 7 and Figure 9). 

Application of a modular contact profilometer enabled the determination of the surface roughness of the specimens, which are presented in Table 3. On the basis of the presented results, it could be stated that the roughness of the samples after the sandblasting process is lower than those before sandblasting. This is the effect of the smoothing surface of the honeycomb structures, which remove the unmelted powder particles adhesively bonded with them. Independent of the cell size, all sandblasted samples are characterized by a statistically comparable level of roughness (Table 3).

### 3.2. Evaluation of the Microstructure and Mechanical Properties of Ti6Al4V

**Figure 10:**
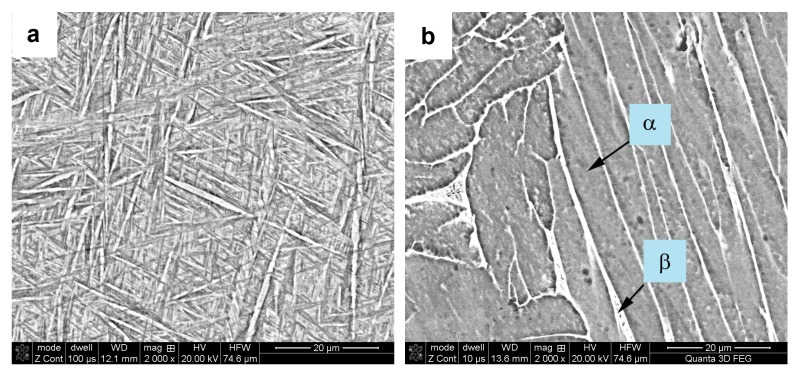
The SEM micrographs of honeycomb components microstructure before (**a**) and after (**b**) heat treatment (1050 °C/2 h).

The next stage of the investigations was related to the evaluation of the microstructure of the Ti6Al4V material. For this purpose, the surface of metallographic samples cut from honeycomb components with or without heat treatment (1050 °C/2 h) was prepared by grinding, polishing and etching with Kroll’s reagent. The microstructure of the samples was examined by a FEI Quanta 3D (FEI company, Hillsboro, USA) scanning electron microscope (SEM) equipped with energy dispersive spectroscopy (EDS). Based on compared SEM photographs presented in Figure 10, it is possible to state that the structure revealed in the sample without annealing consists only of the martensitic phase, which is a Ti-based solid solution (non-equilibrium phase). This phase is formed in titanium alloys during very fast cooling from the temperature area above the transformation α→β temperature. Such conditions of high supercooling occur during the LENS building process. Whereas the microstructure of sample manufactured by LENS technique and heat treated consists of two phases α + β, which were described and confirmed in a previous work [56].

**Figure 11:**
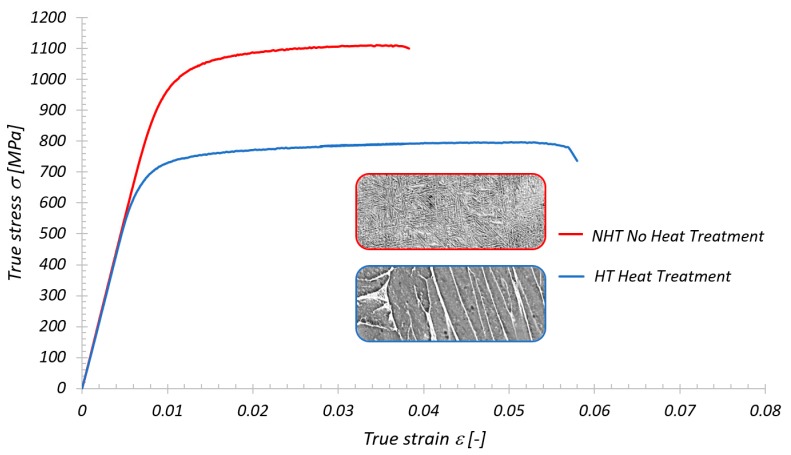
An example of stretching curves for samples cut from the Ti6Al4V thin walls obtained using the LENS technique without (NHT) and with (HT) additional heat treatment process [56].

The mechanical properties of additively manufactured Ti6Al4V materials were determined during a uniaxial tensile test under quasi-static loading conditions. The typical dog bone specimens with a thickness of 0.7 ± 0.1 mm were manufactured with the same technological parameters as the structure specimens. Tensile tests were performed on a MTS Criterion C45 testing machine (MTS Systems Corporation, Eden Prairie, USA), which gives a strain rate of the magnitude 1.7 × 10 s^−1^. The entire process was monitored and recorded using TW-Elite software (ver. 2.3.1, MTS Systems Corporation, Eden Prairie, USA). Figure 11 presents the results obtained for the origin (NHT) and modified (HT) specimens during the heat treatment process. The characteristic mechanical properties of the Ti6Al4V material are presented in Table 4. 

### 3.3. Compression Tests under Quasi-Static Loading Conditions

The mechanical response of regular cellular structures with honeycomb topology was determined during experimental uniaxial compression tests performed under quasi-static and dynamic loading conditions. In both cases, the study structures were placed in an in-plane direction. The obtained results are presented below.

**Figure 12:**
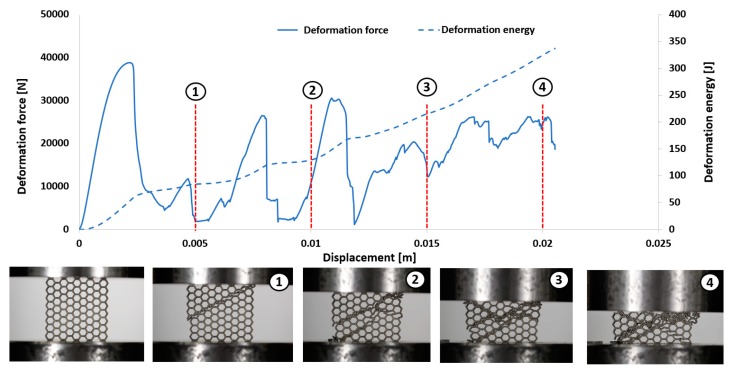
Deformation process of specimen No. 1 manufactured additively with LENS (1–4 stages of deformation during static compression test).

**Figure 13:**
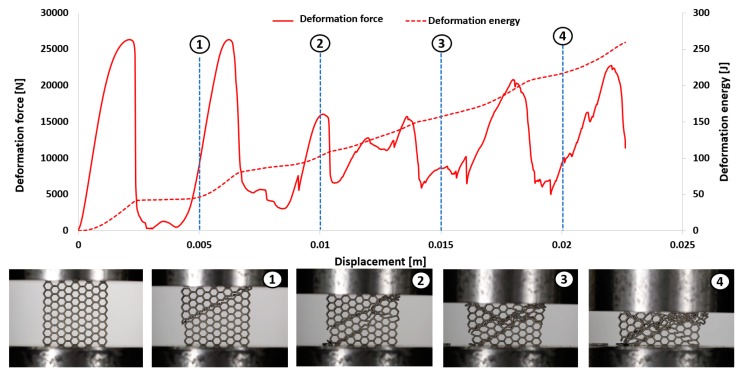
Deformation process of specimen No. 2 manufactured additively with LENS (1–4 stages of deformation during static compression test).

**Figure 14:**
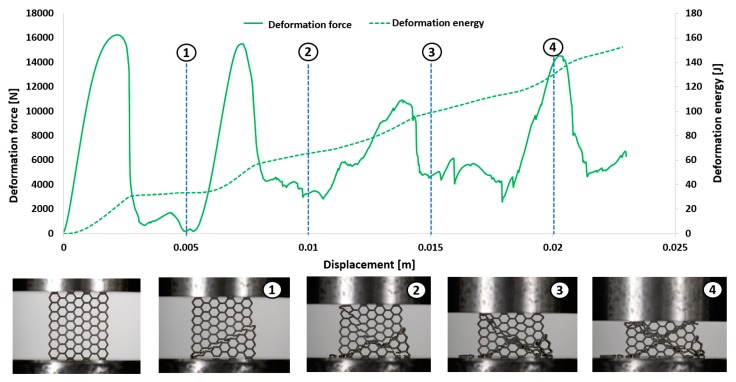
Deformation process of specimen No. 3 manufactured additively with LENS (1–4 stages of deformation during static compression test).

**Figure 15:**
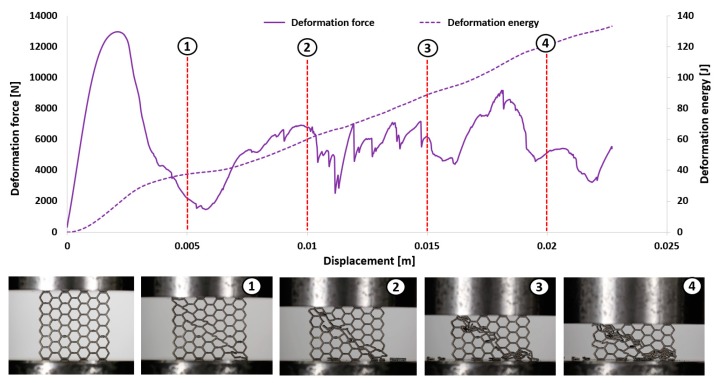
Deformation process of specimen No. 4 manufactured additively with LENS (1–4 stages of deformation during static compression test).

**Figure 16:**
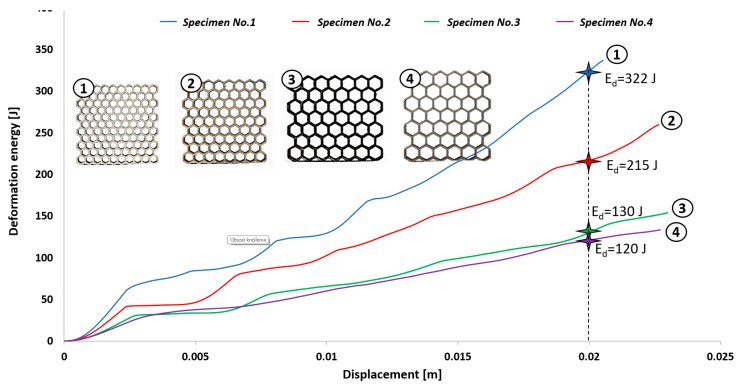
Comparison of deformation energy curves related to structure unit cell size (1–4—the thin-walled honeycomb structures with the different cell size).

The first stage of the investigations was conducted with the use of an MTS Criterion C45 universal tensile test machine and TW-Elite software, which recorded the history of the deformation process. The specimens were placed in the in-plane direction and compressed with 1 mm/s velocity. On the basis of the conducted tests, plots of the deformation force and deformation energy were defined (Figure 12, Figure 13, Figure 14 and Figure 15). From these plots, it was possible to define the relationship between the structure’s relative density when referring to the energy absorption capacity (Figure 16). 

Analyzing the history of the deformation force plots, it could be stated that specimens No. 1 (honeycomb_3) (Figure 12) and No. 2 (honeycomb_4) (Figure 13) demonstrate a high value of the maximum deformation force due to the high geometrical stiffness of the structures of the specimens. The slope of the first part of the historical plots connected with the local buckling effect is similar and caused due to the mechanical properties of the applied Ti6Al4V material and the friction between the surfaces of the specimen and the grip of the testing machine. Depending on the applied elementary unit cell size, the maximum value of the respective deformation force is different. Structures with the lowest unit cell size and highest relative density indicated the highest value of the deformation force. After the buckling effects, a failure mechanism was very quickly achieved. It was mainly caused by the shearing and cracking of the structure walls. The higher value of a unit cell size is represented by specimen No. 3 (honeycomb_5) (Figure 14) and specimen No. 4 (honeycomb_6) (Figure 15), which affects the lower geometrical stiffness of the structures. The maximum value of the deformation force is relatively lower in comparison to specimens No. 1 and No. 2. Moreover, the buckling effect and loss of structural stability appeared later, which caused a delay in the failure mechanism. The plot of the force deformation is milder in this case. The number of the local maximum deformation force is lowest and average (Figure 15). Additionally, the chart presented in Figure 16 demonstrates the relationship between the deformation energy of the specimen referring to the deformation. It could be observed that the higher geometrical stiffness of the specimen caused an increasing maximum value of the deformation energy and caused the reduction of the specimen shortening. Application of topology with a higher value of unit cell size (with lower value of relative density) shows that the maximum value of deformation energy is significantly lower, but the range of the specimen shortening is relatively higher. The value of the deformation energy obtained for the same shortening of all structure specimens (the value of shortening was 20 mm) is presented in Table 5. Based on the presented results, it could be seen that the deformation energy depends on the value of the relative density. Nevertheless, this relationship is not linear, which means that it could also be dependent on the friction process between collaborating elements and the geometrical quality of the additively manufactured structure specimens. Due to having the lowest value of unit cell size, specimen No. 1 was rougher, and the dimensional deviations were higher in comparison to the other specimens. 

### 3.4. Compression Tests under Dynamic Loading Conditions

**Figure 17:**
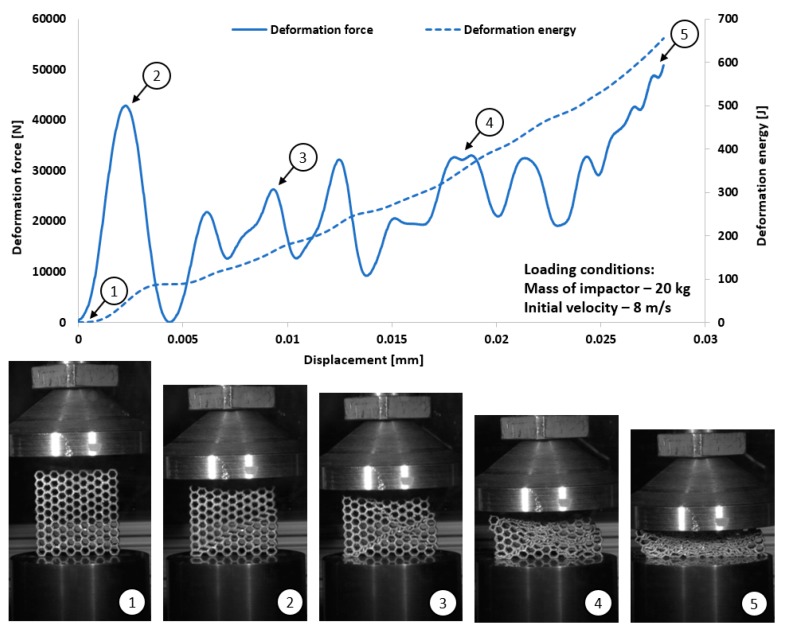
The results of dynamic tests obtained for honeycomb specimen No. 1 (1–5 stages of deformation during dynamic compression test).

**Figure 18:**
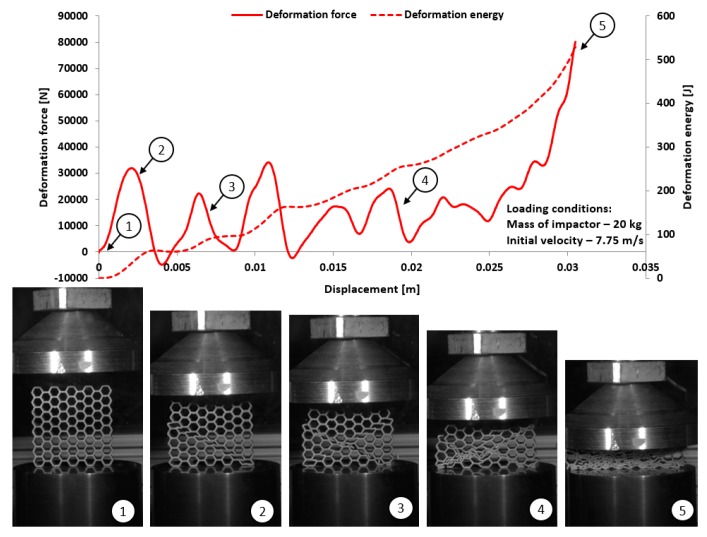
The results of dynamic tests obtained for honeycomb specimen No. 2 (1–5 stages of deformation during dynamic compression test).

**Figure 19:**
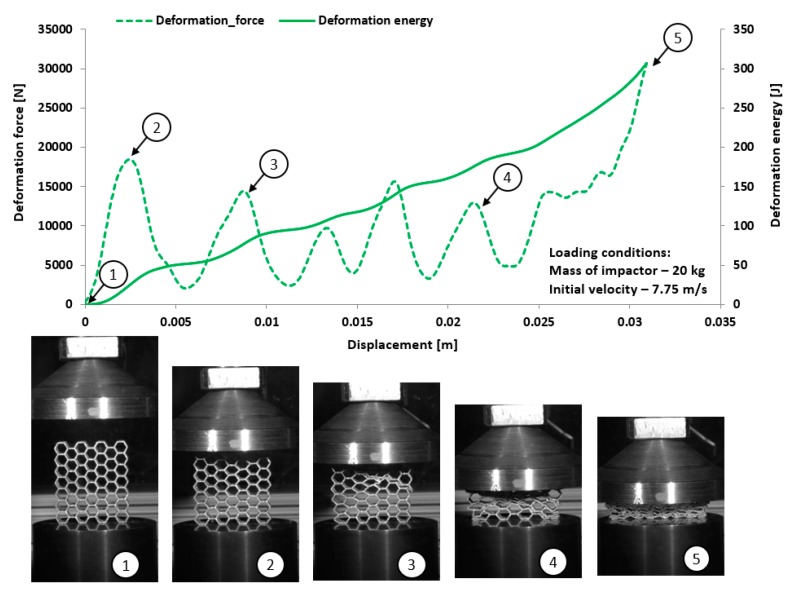
The results of dynamic tests obtained for honeycomb specimen No. 3 (1–5 stages of deformation during dynamic compression test).

**Figure 20:**
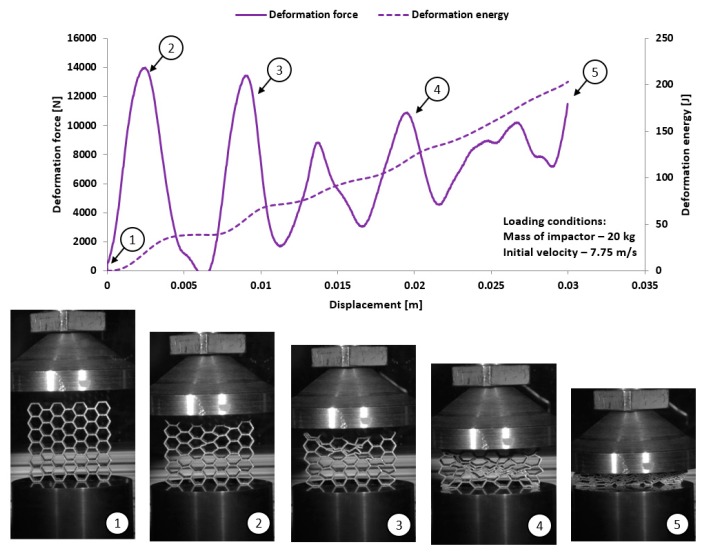
The results of dynamic tests obtained for honeycomb specimen No. 4 (1–5 stages of deformation during dynamic compression test).

The dynamic tests were the other stage of investigations conducted on the mechanical response of additively manufactured Ti6Al4V regular cellular structures with honeycomb topology under dynamic loading conditions. They were carried out with the use of the universal column impact test machine Instron Dynatup 9250 HV with an additional system of data acquisition and a high-speed camera. Adopted initial loading conditions were defined based on the mass of the impactor and its initial velocity. The following initial boundary conditions were used for the tests: impactor mass was 8 kg, and its velocity was 20 m/s. On the basis of the conducted compression tests, the results presented in Figure 17, Figure 18, Figure 19 and Figure 20 were obtained. Analyzing the presented deformation energy plots, it could be stated that the maximum value of the absorbed energy is related to the relative density of the structure of the specimens. The structure of specimen No. 1, with the smallest elementary unit cell size, indicates a higher value of relative density and geometrical stiffness. These features caused the value of the absorbed energy to be significantly higher in comparison to other specimens. Additionally, due to the considerably greater number of cells in the structural arrays, the number of the local maximum deformation force arrived with more frequency. The adopted dynamic initial boundary conditions caused the mechanism of the structural densification to appear for testing cases. 

Comparing the maximum values of the deformation plots in the first stage of the deformation process before the buckling effect, it is possible to observe that the values are relatively higher for quasi-static loading conditions, and they are also contingent on the relative density. The highest value of the deformation force was achieved for specimen No. 1, and the lowest value was achieved for specimen No. 4. Presented in Figure 18 are the results obtained for specimen No. 2, which indicate a negative value of the deformation force after being the first maximum. This phenomenon suggests that after the densification of the first row of structure cells, the rapture damage mechanism caused a spring-back effect of the impactor mass. This evidence was observed in specimens No. 2 and No. 4. The main reason to justify this situation is the presence of local material defects such as microcracks or pores. The presence of the maximum local deformation force could be related to the number of elementary cell rows in the structure. Specimens with the lowest unit cell size demonstrate a more variable plot of deformation force history, which is contrary to a specimen with a larger value of unit cell size. Moreover, the process of structural densification is smoother because the damaged rows of the array do not affect the force deformation history, as in the case of specimens No. 1 or No. 2. 

Considering the dynamic character of the interaction between the specimen and the impactor, it could be observed that the structures with the highest value of the relative density are stiffer and the range of deformation is lower, even when the same impact loading conditions were applied. 

**Figure 21:**
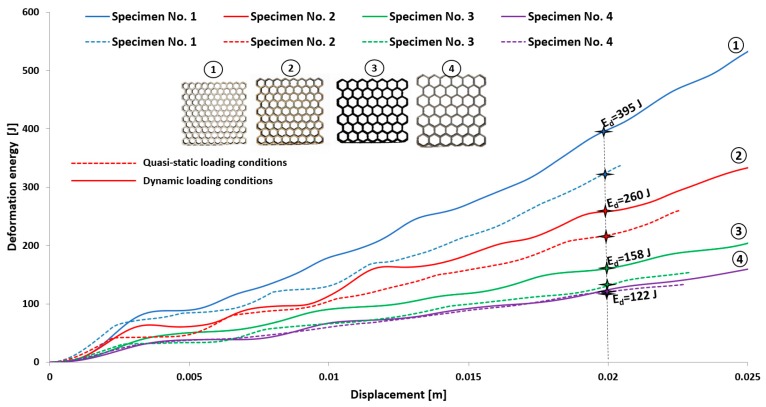
The comparison of deformation energy plots between dynamic and quasi-static results (1–4—the thin-walled honeycomb structures with the different cell size).

The other aspect worth discussing is the deformation rate sensitivity. Comparing the quasi-static and dynamic results, it can be observed that specimens No. 1 and No. 2 demonstrate a high deformation rate sensitivity. Due to the high mass of the structure, the values of the deformation energy are significantly higher in comparison to the results of the quasi-static tests. The low values of relative density and the lowest structural stiffness caused specimens No. 3 and No. 4 to be less and almost insensitive to deformation rate effects. The comparison between the results is presented in Figure 21 and in Table 6. The results obtained in the quasi-static compression tests are marked by dotted lines, and those for the dynamic tests are defined by continuous lines. 

**Figure 22:**
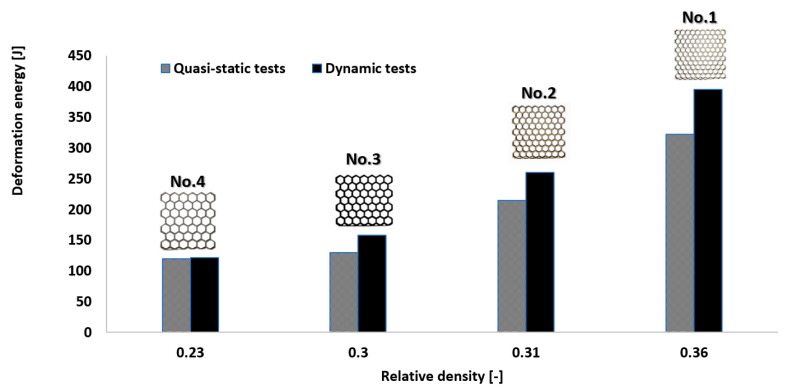
The influence of relative density on structural deformation process under static and dynamic loading conditions.

Figure 22 presents the deformation rate sensitivity of honeycomb specimens versus the various values of relative density. Based on the obtained data, the value of the deformation energy depends on the relative density which is very important from the application point of view. It determines the mass of object and also the effects on its geometrical stiffness. Application of structures with the higher value of relative density causes increasing value of the impact force in the initial stage of structure deformation. Moreover, afterwards the process of structure deformation is more rapid and more destructive due to arriving of the damage mechanism (cracking). Structures with the lower value of the relative density indicate a more smooth deformation history plot. This feature is mostly caused due to buckling and bending mechanisms which preceded the cracking mechanism. Considering the dynamic response of the structure specimen it could be stated that it strongly depends on the value of relative density. Higher values of relative density (specimen No. 1 and No. 2) cause the increasing value of the deformation energy. This phenomenon is generally caused due to the higher mass of specimens and results from inertia effects. Application of lower values of the relative density (Specimen No. 3 and No. 4) allows a reduction in the mass of the object and enables minimization of the effects of impact force in the initial stage of the deformation process. Considering the possibility of honeycomb structures application as a proposal dedicated to energy absorption solutions it is recommended to use the specimens with the lower value of relative density merged with other diverse solid materials. This proposal could be used in civilian (automotive, railway) as well as military (passive protective systems) applications.

## 4. Conclusions

The main aim of this investigation was to analyze the mechanical response of additively manufactured regular cellular structures with a honeycomb topology manufactured additively from a Ti6Al4V titanium alloy with the use of a Laser Engineering Net Shaping system under static and dynamic loading conditions. Based on obtained experimental results following conlcusions are listed:

1. Geometrical assumptions were adopted during the specimen design process that considered the technological possibilities of the used additive manufacturing system. Moreover, the specimens were designed as cuboid elements with similar dimensions and wall thicknesses.
2. Based on the additional heat treatment process that was conducted, it was revealed that the applied Ti6Al4V titanium alloy materials require a heat treatment process in order to improve the mechanical properties of the material (increase of ductility) and stress relief annealing. As a result, the higher range of Ti6Al4V titanium alloy plastic deformation allowed for increasing the structure specimen’s energy absorption capacity. Furthermore, it enabled reduction of the destructive effect of material brittle damage, which is essential referring to safety issues.
3. Uniaxial tests of structural specimens were performed under both static and dynamic loading conditions, which allowed for the evaluation of the specimens’ energy absorption capacity and the sensitivity of the developed specimens on the strain rate. Based on the obtained results, it could be stated that an increasing value of relative density causes a growing sensitivity of the structure for strain rate effects.

## Figures and Tables

**Table 1:** Relative density of the developed honeycomb structures.

Specimen	Honeycomb_3	Honeycomb_4	Honeycomb_5	Honeycomb_6
	No. 1	No. 2	No. 3	No. 4
Unit cell size (mm)	3	4	5	6
Relative density (−)	0.36	0.31	0.3	0.23

**Table 2:** Basic parameters used for manufacturing honeycomb structures by the LENS technique.

Laser Power (W)	Powder Feedrate (rpm)	Layer Thickness [mm]	Powder (material)	Substrate (material)
180	9.8	0.3	Ti6Al4V	Ti6Al4V

**Table 3:** Results of the roughness tests of samples manufactured by the LENS technique without heat treatment.

Sandblasting	Honeycomb Structure	Ra (µm)	St. dev.	Rz (µm)	St. dev.
Before	No. 4	28.66	2.62	170.59	14.83
	No. 3	29.19	1.89	173.97	15.56
	No. 2	27.89	1.98	163.71	17.48
	No. 1	28.26	1.32	166.45	16.78
After	No. 4	22.19	2.74	133.76	7.29
	No. 3	24.52	1.77	140.58	10.42
	No. 2	21.34	2.59	127.77	15.77
	No. 1	22.99	0.70	143.95	13.43

**Table 4:** Strength parameters determined in a tensile test for samples cut from Ti6Al4V thin walls obtained by the LENS technique (before and after the heat treatment process).

Specimen Sample	R _0.2_ (MPa)	Rm (MPa)	A (%)	E (GPa)
NHT	988	1110	3.7	110
HT	705	794	5.6	108

**Table 5:** The comparison of maximum values of absorbed energy referring to honeycomb structure specimens.

	No. 1	No. 2	No. 3	No. 4
Relative density (−)	0.36	0.31	0.3	0.23
Max. value of absorbed energy (J)	322	215	130	120

**Table 6:** The comparison of the maximum values of absorbed energy referring to honeycomb structure specimens.

	No. 1	No. 2	No. 3	No. 4
Relative density (−)	0.36	0.31	0.3	0.23
Max. value of absorbed energy in quasi-static (J)	322	215	130	120
Max. value of absorbed energy in dynamic (J)	395	260	158	122
Average increase of absorbed energy	22.6%	20.1%	21.5%	1.6%
